# Implementation of misoprostol for postabortion care in Kenya and Uganda: a qualitative evaluation

**DOI:** 10.3402/gha.v6i0.19649

**Published:** 2013-04-24

**Authors:** Joachim Osur, Traci L. Baird, Brooke A. Levandowski, Emily Jackson, Daniel Murokora

**Affiliations:** 1 Ipas African Alliance, Nairobi, Kenya; 2 Ipas, Chapel Hill, NC, USA; 3 Independent Consultant, Los Angeles, CA, USA; 4 Uganda Women's Health Initiative, Kampala, Uganda

**Keywords:** misoprostol, postabortion care, implementation research

## Abstract

**Objective:**

Evaluate implementation of misoprostol for postabortion care (MPAC) in two African countries.

**Design:**

Qualitative, program evaluation.

**Setting:**

Twenty-five public and private health facilities in Rift Valley Province, Kenya, and Kampala Province, Uganda.

**Sample:**

Forty-five MPAC providers, health facility managers, Ministry of Health officials, and non-governmental (NGO) staff involved in program implementation.

**Methods and main outcome measures:**

In both countries, the Ministry of Health, local health centers and hospitals, and NGO staff developed evidence-based service delivery protocols to introduce MPAC in selected facilities; implementation extended from January 2009 to October 2010. Semi-structured, in-depth interviews evaluated the implementation process, identified supportive and inhibitive policies for implementation, elicited lessons learned during the process, and assessed provider satisfaction and providers’ impressions of client satisfaction with MPAC. Project reports were also reviewed.

**Results:**

In both countries, MPAC was easy to use, and freed up provider time and health facility resources traditionally necessary for provision of PAC with uterine aspiration. On-going support of providers following training ensured high quality of care. Providers perceived that many women preferred MPAC, as they avoided instrumentation of the uterus, hospital admission, cost, and stigma associated with abortion. Appropriate registration of misoprostol for use in the pilot, and maintaining supplies of misoprostol, were significant challenges to service provision. Support from the Ministry of Health was necessary for successful implementation; lack of country-based standards and guidelines for MPAC created challenges.

**Conclusions:**

MPAC is simple, cost-effective and can be readily implemented in settings with high rates of abortion-related mortality.

Unsafe abortion and its complications are a major health risk to women around the world, particularly in developing countries. Global data indicate that, regardless of abortion laws, abortions occur in measurable numbers in all regions of the world; however, legal restrictions on abortion shift the balance of abortion procedures from those that are legal and safe, to those that are unsafe (1, 2). Such is the case in Eastern Africa, where, despite restrictive abortion laws, rates of unsafe abortion are some of the highest in the world at 36 unsafe abortions per 1,000 women aged 15–44 (3). In Kenya and Uganda, two Eastern African countries where abortion has been highly restricted^1^ (4–6), recent estimates of the maternal mortality ratio (MMR) are 530 and 430 per 100,000 live births, respectively (7). As much as 18–35% of these deaths are attributable to unsafe abortion (8, 9). Morbidity related to induced abortion is similarly high in the region; the annual rate of hospitalization for complications related to induced abortion is 10/1,000 women (10). While striking, these figures likely underestimate the true magnitude of morbidity and mortality related to unsafe abortion, given that stigma, fear of legal reprisals, and deaths outside of the hospital contribute to gross under-reporting of abortion-related mortality (1).

Postabortion care (PAC) is an approach for reducing deaths and injuries from incomplete and unsafe abortions by removing the remaining products of conception from the uterus and treating associated complications, such as bleeding or infection. The PAC model also includes provision of post-treatment family planning and links to other needed reproductive health services and to the community (11). The World Health Organization's (WHO) recommended methods of uterine evacuation for PAC in the first trimester of pregnancy are uterine aspiration, with either electric (EVA) or manual vacuum aspirators (MVA), or medical treatment with misoprostol (MPAC) (12).

A growing body of evidence has demonstrated that medical treatment of incomplete abortion with misoprostol is an effective and acceptable alternative to vacuum aspiration. Misoprostol, a prostaglandin-E1 analogue, is employed for multiple obstetric and gynecologic indications, including treatment and prevention of postpartum hemorrhage, induction of abortion and induction of labor, among others (13–19). Misoprostol's ability to induce uterine contractions and soften the cervix makes it similarly effective in emptying the uterus following incomplete abortion (13, 20). In studies involving at least 100 women, misoprostol for incomplete abortion has demonstrated an average efficacy of 95%, with the highest reported success rate being 99%—rates comparable to treatment with MVA (21–23). Because it is inexpensive, easy to use, stable at room temperature, and can be administered by a variety of non-parenteral routes (oral, sublingual, buccal, and vaginal), misoprostol is well suited for use in low-resource settings (24). Safety and efficacy of misoprostol for treatment of incomplete abortion due to both spontaneous abortion/miscarriage and induced abortion have been established by a number of studies conducted in low-resource settings, including Tanzania (22), Mozambique (25), Burkina Faso (26), and Uganda (23), as has women's satisfaction with misoprostol for this indication (22, 23, 25, 26). A recent study documenting the introduction of misoprostol for PAC in Nigeria found that both women and providers were highly satisfied with MPAC, and that integration of misoprostol into clinical services was straightforward and required few resources (27). WHO includes misoprostol in its evidence-based guidelines and recently added it to their Model List of Essential Medicines (28) for medical management of incomplete abortion; misoprostol had been previously included on this list for early pregnancy termination (in combination with mifepristone), and labor induction (29).

We sought to evaluate the implementation of MPAC services in Kenya and Uganda. Study objectives were: (a) to evaluate the process used to introduce MPAC in these two countries, including barriers encountered and facilitating elements; (b) to assess provider satisfaction with MPAC and provider impression of patient satisfaction with MPAC; and (c) to identify supportive and inhibitive policies around the use of MPAC. These data can be used to inform future MPAC programming efforts, both those being newly established and those ready for scaling up.

## Methods

### Study overview

In both countries, a baseline assessment of health facilities participating in the project was conducted prior to the implementation of MPAC services. During implementation, site improvement to address deficiencies identified during the baseline assessment, and health worker training and follow-up support were provided. Trained health care workers provided MPAC for 13–25 months, at which time this qualitative evaluation of the program, utilizing in-depth interviews with program participants, was completed.

This project was designed and implemented by Ipas, an international non-governmental organization (NGO) dedicated to ending preventable deaths and disabilities from unsafe abortion, in partnership with local Ministries of Health.

### Setting

The study was conducted in the Rift Valley Province of Kenya and the Kampala Province of Uganda. Health facilities within these provinces were purposively selected to participate based on demand for PAC, willingness of providers and administrators to support implementation of the pilot program, and availability of necessary infrastructure for service provision. Although the selected health facilities were not intended to be representative of all facilities in the two countries, efforts were made to include both public and private facilities, as well as those operating at the primary, secondary, and tertiary care levels. In Kenya, five public hospitals were included; in Uganda, two public hospitals and 18 clinics belonging to private midwives were included.

### Baseline facility assessment

Prior to the implementation of MPAC, capacity of the participating sites was evaluated utilizing a comprehensive site assessment tool (30). This tool documented an existing caseload for PAC services and evaluated support for the program among staff and facility managers, adequacy of infrastructure and equipment, human resources capacity, and quality of care provided. As MPAC was to be offered as an alternative to PAC with MVA, it was decided that sites should be capable of providing both methods of treatment for incomplete abortion, ensuring a choice of treatment for women; capacity to provide PAC with both MVA and MPAC was strengthened as necessary.

Based on the initial assessment results, facilities in both countries were upgraded to ensure adequate examination rooms for PAC provision, locked cabinets for storage of misoprostol, MVA equipment and post-treatment family planning methods, and record books were introduced for documentation of PAC cases. The process of registration of misoprostol for PAC was initiated, protocols for MPAC provision were developed and disseminated, and training of MPAC providers was begun.

### Program implementation

**Fig. 1:**
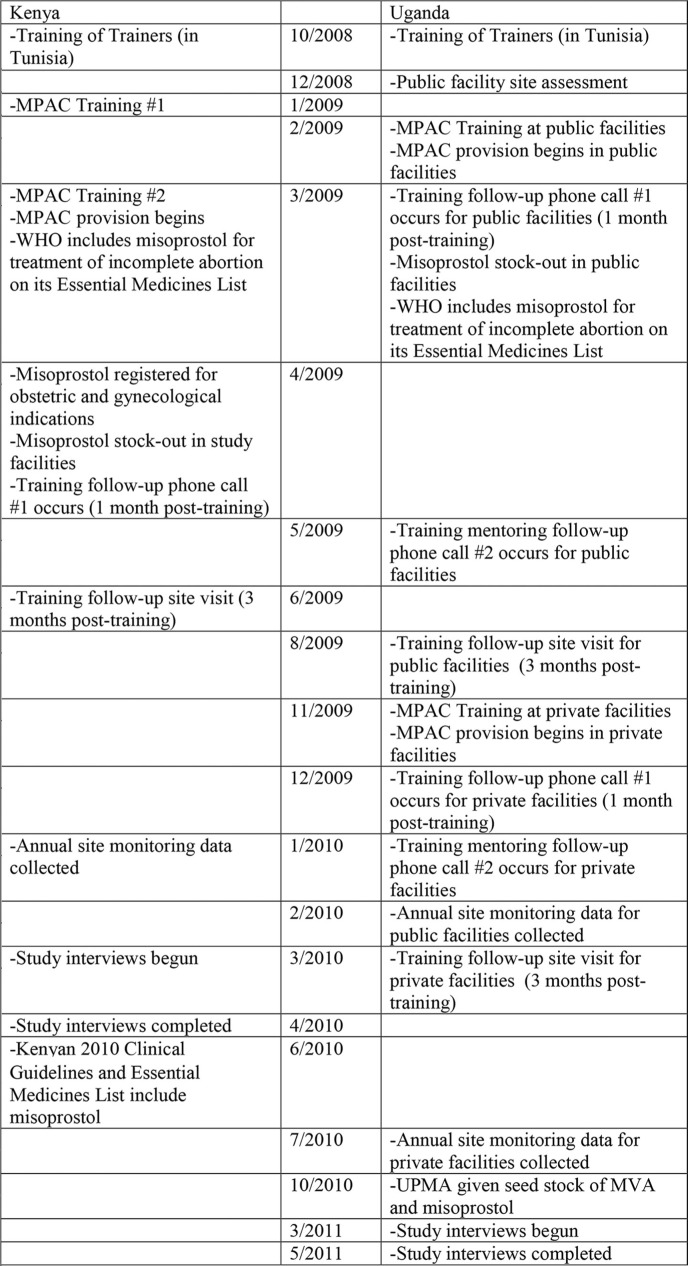
Timeline for implementation of MPAC in Kenya and Uganda.

In both Kenya and Uganda, implementation of the MPAC program was a multi-step process extending over several years (Fig. 1). As an initial step, representatives from both countries attended a 7-day regional medical abortion and MPAC ‘Training of Trainers’ workshop in Tunisia in 2008. This workshop consisted of values clarification exercises,^2^ training in the provision of medicines for uterine evacuation, and clinical site visits to observe use of medical uterine evacuation in the health care setting. Following this training, pilot programs to introduce MPAC into Kenyan and Ugandan hospitals and health centers were developed with the respective countries’ Ministries of Health. Service delivery protocols for provision of MPAC were developed for use in both settings (Table 1). Participating sites’ facilities and equipment were upgraded where necessary, and an initial stock of commodities, including misoprostol, was procured for use in the pilot program.

**Table 1:** Clinical protocol for use of misoprostol in PAC (31)

Confirm diagnosis of incomplete or missed abortion:
History consistent with incomplete or missed abortion Physical exam with gravid uterus of less than 12 weeks size and an open cervical os with uterine bleeding (incomplete abortion), or a closed cervical os and confirmation of pregnancy loss by ultrasound (missed abortion)
Evaluate for conditions that require specialized or alternative care:
Contraindications to misoprostol including known allergy to prostaglandins, confirmed or suspected ectopic pregnancy, hemorrhagic disorder or concurrent anticoagulant therapy If an intrauterine device is in place, removal of device prior to administration of misoprostol
Counseling
Emotional support Return to fertility, contraceptive counseling and provision of contraceptives What to expect after using misoprostol Pain, bleeding and expulsion of pregnancy tissue Nausea, vomiting and diarrhea Fever, chills Discuss and provide pain management Review warning signs Excessive bleeding Signs of infection Failure of treatment
Administer misoprostol
Misoprostol 600 mcg orally OR 400 mcg sublingually Administer pain medication simultaneously Let the client wait in facility for 30 min Re-administer if client vomits within 30 min Antibiotics may be used if history and physical are suggestive of infection Where prevalence of Rhesus-negative status is high and Rhesus immunoglobulin is available, Rhesus immunoglobulin may be administered to Rhesus-negative women
Follow-up
Schedule a follow-up visit in 2 weeks Confirm completion of expulsion History: bleeding and cramping with passage of clots and/or tissue Exam: uterus is normal size firm, and non-tender; the adnexae are non-tender; cervical os is closed Review contraception Refer client for other services as needed

In Uganda and Kenya, 51 and 22 health care workers respectively were trained in provision of MPAC (see Table 2). Individuals trained were a mix of obstetrician–gynecologists, medical officers and midwives. The 5-day training included values clarification, training in clinical assessment of the client, use of misoprostol for PAC, MVA for PAC, management of missed abortion and early pregnancy failure, postabortion contraception, infection prevention, management of complications, referral and community involvement. After training, follow-up support continued in both countries. This support consisted of follow-up phone calls every month for the first 3 months made by clinical trainers with additional training and skills in supporting providers to launch and maintain MPAC services, and a site visit 6 months after program implementation. At these visits, records were reviewed and case management was discussed.

**Table 2:** Facilities and providers included in MPAC pilot program

Region	Kenya Rift Valley Province	Uganda Kampala Province
Public facilities	3	2
Private facilities	2	18
Providers trained	22	51

### Evaluation of MPAC implementation

Following implementation of MPAC programs in both countries, an independent evaluation of the implementation process was conducted. In-depth qualitative interviews were conducted with MPAC service providers, heads of the health care centers where MPAC had been introduced, and local policymakers who had helped establish MPAC services. Additionally, Ipas staff and consultants who had been involved in the implementation of MPAC were interviewed.

Four guides, tailored for each type of study participant, were developed for the interviews; the same guides were used in both countries. These guides contained open-ended questions aimed at assessing strengths and weaknesses of the intervention, barriers and facilitating factors encountered during implementation, providers’ satisfaction with MPAC and providers’ impressions of patients’ satisfaction with MPAC. Participants were asked questions specific to their role in the program, as well as general questions about the program and its implementation; data from all types of participants were compiled to provide a summary evaluation of the program. In each country, a single interviewer conducted interviews. Data were collected between March and April 2010 in Kenya, and from March to May 2011 in Uganda. A total of 45 interviews, 15 in Kenya and 30 in Uganda, were completed (Table 3).

**Table 3:** Study participants in Kenya and Uganda

Respondents	Kenya	Uganda
Ministry of health	1	2
Ipas staff	2	2
Health facility managers^a^	5	6
MPAC trainers	2	4
MPAC providers	5	16
Total	15	30

a Health facility managers were also providers; they are reported separately here.

In addition to interviews, the evaluators reviewed reports collected during program implementation, such as site improvement reports, training assessment reports, drug registration and procurement reports, and information related to the availability of misoprostol. These reports provided data related to the process of implementation of the program, such as numbers and types of individuals trained, obstacles encountered when establishing services, and interim work plans. Routinely collected program monitoring data were used to determine number of women served by the project.

### Management and analysis of evaluation data

In Kenya, interviews were recorded and fully or nearly fully transcribed, while in Uganda, interview data was largely captured via interviewer notes; only a small number of interviews were recorded. Three investigators, one in Uganda, one in Kenya, and one who worked on the evaluation in both countries, coded interview data utilizing a framework analysis plan developed prior to the interviews, and reflected in the interview guides. This framework asked participants to comment on the strengths and weaknesses of the program and its implementation, including training and ongoing support, procurement of commodities and equipment, involvement of relevant stakeholders, sustainability of the program and potential for scaling up. Additionally, participants were questioned about any perceived changes in access to PAC services, quality and acceptability of those services. Differences between the two countries are noted; no gender differences in responses of study participants were noted. These data were supplemented with information gleaned from the above-mentioned reports documenting the program implementation, as well as interviewer notes. In both countries, data were collected to the point of saturation, after which no new information emerged during interviews (32). To ensure validity and consistency across study sites and countries, an additional investigator independently reviewed all coded data.

## Results

### Use of PAC services and women served by program

Service delivery data collected from sites at the 6-month follow-up visit were used to estimate the number of women served during the pilot projects (Table 4). In Kenya, 222 women were served in 3 months, leading to an estimated total of 962 women served during the 13 months between when the pilot began and when the evaluation interviews were conducted. Of those, 47% (*n*=455) of women received PAC with MVA and 53% (*n*=507) received MPAC. In Uganda, 25 months elapsed between the beginning of the pilot and conducting the interviews. In that period of time, an estimated 5,467 women were served in public facilities for the full 25 months and private midwives served 949 women for 16 months. The vast majority (87%, *n*=4,758) of women served in public facilities received MPAC, compared with only 39% (*n*=373) of women seen by private midwives.

**Table 4:** Estimated number of women served during the pilot programs

	Kenya	Uganda
Number of women served in 3 months	222	656 – public facilities
		178 – private facilities
		834 – overall
Proportion served with misoprostol	52%	87% – public facilities
		39% – private facilities
		77% – overall
Proportion served with MVA	47%	13% – public facilities
		61% – private facilities
		23% – overall
Number of women served during pilot*	962	5,467 – public facilities
		949 – private facilities
		6,416 – overall

* Kenya: using 3 months of service delivery data annualized to cover 13 months of the intervention, from March 2009 through March 2010; Uganda: using 3 months of service delivery data annualized to cover 25 months of intervention in public facilities, from February 2009 through March 2011 and 16 months of intervention in private facilities, from November 2009 through March 2011.

### Barriers and facilitating factors for implementation of MPAC

#### Enabling environment

In both Kenya and Uganda, the creation of an enabling environment and stakeholder support of MPAC implementation were key to the success of the intervention. Support from key officials in the Ministry of Health, members of local medical boards and ethics committees, and heads of the Obstetrics and Gynecology Departments in the implementing hospitals and clinics facilitated the implementation process and the success of the program. Involving such individuals from the project's inception was helpful in ensuring their continued support:

Our hospital administration were satisfied, and even chose two people from the facility to attend the training. Because they were involved, they have supported us.

MPAC Provider, Kenya

Interviewees acknowledged that to move beyond the pilot phase, broader stakeholder involvement, particularly of international NGOs and multilateral organizations working in maternal health, would be advantageous to ensure continued success of the program.

#### Commodity security

Commodity security presented initial and ongoing challenges in both settings. Initial stocks of misoprostol were procured for use in the study in both countries. In Kenya, however, the implementation of the program was delayed while the drug was formally registered for obstetric and gynecologic indications—a requirement of the Ministry of Health before misoprostol could be used within the confines of the program. Following this registration, local pharmacists were trained in its clinical uses and in properly referring women presenting with incomplete abortion to health care facilities before the pharmacies were supplied with misoprostol from the pharmaceutical distributor. Underestimation of demand for the medication led to stock-outs of the drug within 3 months of initiation of MPAC services, and failure of the involved institutions to allocate a budget to purchase the medication delayed acquisition of misoprostol by approximately 1 month, during which time it was unavailable. In Uganda, where misoprostol had previously been registered for use in treating postpartum hemorrhage, Ministry of Health officials initially expressed concern about ‘off-label’ use to treat incomplete abortion. Eventually, the Ministry of Health approved use of misoprostol for PAC within the confines of the program. However, they did not develop a policy for sustained procurement, leading the two Ugandan public health care facilities to experience stock-outs of misoprostol within 1 month of implementation. Stores of the medication intended for use in postpartum hemorrhage were not allowed to be re-allocated for MPAC:

The major challenge is for the Ministry of Health to bring out a policy to address [misoprostol] usage as one of the effective drugs … in maternal health care. The National Medical Stores should stock the drug, and hospitals and other facilities should have proper records and requisition procedures for their stocks.

Ministry of Health Official, Uganda

Providers interviewed in both countries believed that a formal agreement between program implementers and the facilities where the protocol was implemented–such as a Memorandum of Understanding–outlining a plan to maintain supplies of misoprostol and delineating responsibility for such maintenance, would have avoided the observed stock-outs. The private UPMA facilities in Uganda included in the pilot program received initial stocks of misoprostol through the project and developed a procurement plan for resupply, and did not experience the commodity stock-outs observed in public facilities in both Kenya and Uganda. At these facilities, the overarching UPMA organization provided misoprostol to their member facilities at a reasonable price. These funds were then used to purchase additional supplies of misoprostol as well as subsidize the operational costs of the organization. Monthly training meetings provided a forum for health care providers in the individual clinics to replenish their misoprostol supplies.

#### Facility/staffing requirements

Beyond ensuring commodity security, study participants in both countries agreed that facility and staff requirements for provision of PAC were minimal. Public facilities in both Kenya and Uganda had a higher volume of patients requiring services than the private facilities included in the pilot. In these busier settings, providers recommended establishing two rooms for PAC provision, and that each room be fitted with a lockable cupboard in which to store medications and supplies and an examination/procedure table; two rooms was considered ideal to facilitate patient flow. In facilities with a lighter caseload, one room was considered adequate for counseling and performance of PAC with MVA or misoprostol.

In busier facilities, participants estimated that a minimum of one to two MPAC-trained providers should be available at all times. Given frequent absences of clinical staff, such as for continuing medical education activities, and the need to allow for routine rotations to other wards, staff days off, and day and night duties, it was suggested that program managers consider these varying schedules to ensure adequate presence of trained providers. On-the-job training in MPAC and avoidance of transfers or unnecessary rotation of trained staff to other locations or departments were offered by study participants as two strategies to ensure sustainability of trained staff. In smaller facilities with fewer cases, one provider was adequate to meet patient demand.

#### Training

In both countries, study participants complimented the training that they had received for MPAC. Follow-up mentoring of trainees, via phone calls and in person, provided support to new MPAC providers and was valuable in ensuring high quality of care.

### Provider perspectives of MPAC

#### Workload decompression

In both Kenya and Uganda, providers were very satisfied with MPAC, and perceived that their clients were similarly satisfied. Because the space and equipment requirements for MPAC are minimal, and midlevel health care providers in both countries were permitted to provide MPAC, implementation of MPAC decompressed the workload of PAC providers and decreased demand for those health facility resources at higher levels in the health care system, such as hospital beds, procedure rooms/operating theaters, and MVA equipment, necessary to provide PAC with MVA:

Previously, when it was MVA alone, some patients used to stay here for one or two days waiting for MVA because if you have only two or three MVA kits, you have to do [the procedure], you process the equipment … sometimes we have a lot of patients. …. So [MPAC] has made it easier, more accessible to the patients, and also to the providers.

Health facility manager and MPAC provider, Kenya

It actually increased on the options for treatment and was embraced by the health workers who were prescribing misoprostol … The fact that there was reduced surgical intervention led to a reduction in patient load and time of waiting.

MPAC provider, Uganda

Midlevel providers effectively administered MPAC, reporting decreased numbers of women awaiting treatment with MVA, and freed skilled staff, including midwives, to participate in other health care activities, particularly those requiring a higher level of training. For those providers trained in both MPAC and MVA, MPAC was seen as easier and less time-intensive:

I am on night duty … and it can be very busy … When I'm doing my rounds, I give [patients] misoprostol and continue with other work … I cannot do the same with MVA—attend to other patients at the same time. So, it is easier for me to give the misoprostol than to do the MVA.

MPAC provider, Kenya

#### Benefits for patients

Providers stated that misoprostol appeared to be very effective and associated with few complications. Providers reported difficulty in getting women to return for a recommended follow-up appointment after misoprostol use; some attributed poor follow-up rates to MPAC's efficacy:

The drug is very effective … on those with incomplete abortion … There are those who will come back after a week, but most of them do not usually come back to give us feedback …. The ones who have problems will always come back. That's why when they do not come back, we assume they are well.

MPAC provider, Kenya

Providers believed MPAC caused women less discomfort than PAC with MVA, and, as it does not necessitate instrumentation of the uterus, that MPAC carried a lower risk of postabortal infection. Oral administration of misoprostol for PAC allowed women to avoid both a procedure and admission to the hospital:

Since it gave them [clients] a viable and much less painful method or option … many of the clients were satisfied. Many fear instrumentation requiring taking them to the theatre, which sometimes requires giving them anesthesia or some kind of sedation. So all these would be avoided where you use misoprostol.

MPAC provider, Uganda

Providers also believed misoprostol to be more affordable for women, both because the treatment itself is inexpensive and because it avoided the additional expenses associated with a surgical procedure and hospital admission. Avoiding an inpatient stay additionally offered women greater confidentiality:

Abortion, including miscarriages, are much stigmatized in this country and also in the communities where these women come from. So, when they are given the drug and go home, nobody gets to know the treatment they are getting. They don't need to worry about answering so many questions as to why they have been admitted to the hospital.

Program staff, Kenya

However, in both countries, providers noted that a subset of women, particularly younger women, preferred MVA to misoprostol for PAC. They believed that these women desired a more efficient treatment, and the knowledge that their treatment was complete before leaving the health facility and returning home. Some providers themselves expressed concern with the uncertainty associated with misoprostol use:

With misoprostol, you administer it but you are still not very sure it will complete the abortion or the woman will come back with prolonged bleeding … sometimes it doesn't remove the products completely, so you end up again doing MVA … MVA's advantage is that you are sure you have removed [the products of conception] and the bleeding stops instantly.

MPAC provider, Kenya

### Supportive and inhibitive policies for implementation of MPAC

#### Increased focus on maternal mortality

In both Kenya and Uganda, participants stated that a desire to address maternal mortality due to unsafe abortion opened the door to provision of MPAC. At the time of the pilot, the Kenyan Ministry of Public Health and Sanitation had officially adopted PAC as a means to reduce maternal mortality; as such, strategies to scale-up PAC, particularly cost-effective interventions such as MPAC, were considered welcome additions. Similarly, Uganda had prioritized maternal mortality in its Road Map for Reduction of Maternal and Neonatal Mortality (33) and its National Development Plan (34).

#### Importance of visible country support

Participants cited lack of country-based standards and guidelines for MPAC in either Kenya or Uganda as a challenge to implementation of MPAC services. Although recommendations from WHO and NGOs were available, study participants stated that national standards were necessary, not only to provide a locally approved framework for provision, but also to codify governmental support for use of the technology. In Kenya, attempts to include misoprostol in national guidelines for PAC had met with resistance, despite its proven benefits. Government ownership of MPAC programs, as evidenced by integration into national policies, could facilitate scaling up of successful pilot programs.

Registration status of misoprostol was an important consideration in both settings. In Uganda, where misoprostol was registered for postpartum hemorrhage at the time of the program implementation, difficulties in procurement of the drug specifically for use in MPAC, outside of its registration, led to temporary stock-outs of the medication within the program. An application for registration of misoprostol for treatment of incomplete abortion in Uganda was approved after completion of the pilot program. Broad registration of misoprostol in Kenya was necessary and achieved before the Ministry of Health approved the implementation of the MPAC project in that country. Inclusion of misoprostol on WHO's List of Essential Medicines for labor induction and medical abortion (35) at the time of program development and implementation lent credibility to proposals to utilize misoprostol for reproductive health indications, despite the fact that misoprostol was not added to the WHO list for incomplete abortion until 2009 (29). Soon after completion of the pilot program in Kenya, misoprostol was added to the Kenyan Essential Medicines List for management of incomplete abortion (36).

Policies dedicated to MPAC, when implemented, helped to facilitate dedication of health care resources for ongoing MPAC training and supplies necessary to maintain programs. Regardless of registration status of misoprostol, lack of dedicated funding and a secure supply chain for MPAC commodities led to stock-outs of the medication during program implementation in both countries. Reliance on donations of misoprostol, as well as for other PAC commodities such as MVA kits, impeded reliability and sustainability of these programs.

## Discussion

Evaluation of this pilot MPAC program in two African countries illustrates a number of facility factors and policy inputs that facilitate the successful implementation of MPAC services. To our knowledge, this is the first study assessing the implementation of an MPAC program outside of a clinical trial; these data can be used to inform future MPAC programming efforts, both those being newly established and those ready for scaling up. We found that providers, health center administrators and policy makers were very satisfied with the MPAC program, and perceived that women receiving MPAC services were satisfied as well. Earlier studies have established the acceptability of MPAC to women for the treatment of incomplete abortion (22, 23, 25, 26). Stated benefits of the program for health care facilities included task shifting of MPAC provision to midlevel providers, with resultant workload decompression for those health care workers providing MVA services; this sentiment was expressed even though midlevel providers were trained to offer both misoprostol and MVA for PAC in this project. These findings echo those of a recent Nigerian study assessing provider acceptability of MPAC (27). Further, provision of routine PAC services, both MPAC and MVA, by midlevel providers, freed those with a higher level of training to attend to more complicated cases. Respondents appreciated the decreased demand for hospital resources, particularly for hospital beds, operating theatre/procedure room time, and equipment as a result of MPAC implementation. Additionally, MPAC providers believed that use of misoprostol offered women greater privacy, reduced cost, and the option of a non-invasive treatment for their incomplete abortion.

An enabling environment, with support from the Ministry of Health and hospital administrators, was an important precondition to establishing a successful MPAC program. However, lack of national policies and guidelines for MPAC created barriers to its implementation. Integration of MPAC into national health care policies will both lend legitimacy to the use of misoprostol for PAC, and help to secure financial and other forms of health systems supports for MPAC service provision. The importance of governmental buy-in when expanding abortion care in legally restricted settings has been previously established (37).

Given the limited resources necessary for MPAC, implementation of MPAC programs was seen as achievable in many settings. Evaluation participants commented that adequate training in misoprostol use was necessary for successful implementation, and that follow-up support after training was helpful in ensuring appropriate misoprostol provision and service quality, in addition to providing support to health care providers engaged in the MPAC program. A recent, large randomized trial comparing MPAC to PAC with MVA in five sub-Saharan African countries similarly recommends adequate provider training with subsequent follow-up and support as a means to reduce unnecessary interventions while new providers become familiar with the amount and duration of misoprostol side-effects, particularly bleeding (38). In those health care settings where maintaining a pool of adequately trained providers, and ensuring that trained providers were available at all times, presented challenges, pre-service training, on-the-job training, and minimizing loss of trained providers are recommended.

In both Kenya and Uganda, procuring misoprostol and maintaining adequate supplies of the drug was difficult. Registration of misoprostol for the treatment of incomplete abortion, accurately estimating demand for misoprostol, and creating a formal plan to maintain supplies of misoprostol with clearly delineated responsibilities for such maintenance, were solutions proposed by study respondents to prevent commodity stock-outs. In Kenya, misoprostol was broadly registered for obstetric and gynecologic indications as a precondition of program implementation; in Uganda, misoprostol was registered in 2011 for use in incomplete abortion as well as for other indications (39) after the pilot program concluded. These advances should facilitate future MPAC projects and scaling up of this pilot program. In response to difficulties in estimating misoprostol needs, Ipas has developed a tool to assist health facilities in determining quantities of medications, including misoprostol, that they will likely need when implementing and maintaining MPAC and medical abortion programs (40).

This pilot evaluation suffers from several limitations. Importantly, as this study was designed as an evaluation of the process of implementation of MPAC pilot programs in both Kenya and Uganda, baseline service delivery data prior to implementation and client satisfaction data from women receiving MPAC services were not collected. Although baseline data were not collected, MPAC was unavailable (outside of a research context) prior to this pilot program in both countries. Studies assessing women's satisfaction with MPAC have been previously published (22, 23, 25, 26); however, data specific to the Kenya and Uganda settings may be helpful in improving those countries’ services. Women were not followed in this study, so we cannot substantiate providers’ views that women who did not return for follow-up were satisfied with MPAC and had no need for additional care. As the pilot was implemented in a limited number of facilities that had shown in interest in providing MPAC services and did not include a control group, the highly positive attitude of our study participants toward the program and MPAC in general may not be observed in other facilities. Additionally, it is possible that social desirability influenced the positive responses of the participants, particularly given that values clarification exercises were included in the program and that administrative support for the program was a requirement for facility inclusion. We did not question participants regarding their attitudes toward MPAC or incomplete abortion in general, but gathered data related to program implementation and provision of MPAC services. Due to the design of the intervention, we did not assess access to or use of misoprostol for PAC outside of formal health facilities. Future projects and studies would ideally incorporate community outreach workers, village health workers, or pharmacy workers with regard to their roles in increasing women's access to PAC overall, and misoprostol specifically.

Despite these limitations, valuable information was gathered about the implementation of misoprostol for PAC in these two low-resource settings. Information was collected from agency staff, Ministry of Health officials, and providers, leading to multiple layers of overlapping information. This informative detail will be used to adapt future introductions of misoprostol in varying settings.

## Conclusion

Although MPAC is a simple, cost-effective and scientifically proven intervention, services are not available where needed—as is the case in many parts of Africa. This program evaluation highlights a number of facilitating factors, such as an enabling environment, adequate training and ongoing support of providers, ease of provision of MPAC, and linking MPAC with maternal mortality reduction goals, for the successful implementation of MPAC. We also identified several pitfalls to avoid, such as failure to establish an ongoing plan for procuring misoprostol, inadequate staffing of facilities with providers trained in MPAC, and failure to incorporate MPAC into governmental policies, including registration of the drug specifically for PAC. We hope this evaluation will assist governments, NGOs, health care providers, and others to effectively implement MPAC programs, and ultimately save women's lives.

## Footnotes

1 Kenya adopted a new constitution in 2010 (6), which has reduced restrictions on abortion. However, unsafe abortion remains a problem in the country.

2 Values clarification exercises offer participants a safe space to explore, question, clarify and affirm their values and beliefs about abortion and related sexual and reproductive health issues. These activities engage stakeholders to identify and address barriers to abortion service provision, access and quality of care stemming from misinformation, values conflict, negative attitudes and lack of respect for women's rights.
